# Optical coherence tomography angiography findings in patients with acute nonarteritic central retinal artery occlusion treated with carbogen inhalation

**DOI:** 10.1097/MD.0000000000046129

**Published:** 2025-11-21

**Authors:** Pongrapee Atipas, Patama Bhurayanontachai, Pichai Jirarattanasopa, Wantanee Dangboon Tsutsumi, Thada Tantisarasart, Mansing Ratanasukon

**Affiliations:** a Department of Ophthalmology, Faculty of Medicine, Prince of Songkla University, Hat Yai, Songkhla Province, Thailand.

**Keywords:** carbogen, central retinal artery occlusion, noninvasive treatment modality, optical coherence tomography angiography

## Abstract

Central retinal artery occlusion (CRAO), a retinal vascular disease that causes severe vision loss, can be evaluated by noninvasive modalities such as optical coherence tomography angiography (OCTA). However, no longitudinal quantitative studies have assessed OCTA in eyes with CRAO treated with carbogen inhalation. In this prospective, observational study, we evaluated the parametric changes of OCTA in patients with acute nonarteritic CRAO treated with carbogen inhalation. Best-corrected visual acuity and all OCTA parameters in eyes with acute nonarteritic CRAO were obtained before initiating carbogen inhalation, 12 to 24 hours and 36 to 48 hours after, and at 1 to 2 weeks and 4 to 6 weeks of follow-up. Twenty-five CRAO eyes were included. The initial OCTA showed reduced vessel density (VD) in both the superficial and deep capillary plexus in the whole-image area, parafovea, and perifovea, which remained decreased until 4 to 6 weeks. The VD at the fovea initially increased, followed by a reduction at 1 to 2 weeks and 4 to 6 weeks. Retinal edema was universal and improved at 1 to 2 weeks and 4 to 6 weeks. No VD recovery was observed after carbogen inhalation. Eyes with acute nonarteritic CRAO showed VD reduction, except at the fovea. No VD recovery was observed after carbogen inhalation. Baseline OCTA parameters did not predict favorable visual outcomes.

## 1. Introduction

Central retinal artery occlusion (CRAO) is a retinal vascular disease that causes severe vision loss, with an incidence of 1 in 100,000.^[1]^ The diagnosis of CRAO is usually made clinically based on, for example, retinal edema, cherry-red spots, box-carring, or attenuation of the vessels.^[2]^ Many noninvasive modalities can be used for diagnosis, such as optical coherence tomography angiography (OCTA). Unlike standard fundus angiography, OCTA does not require the injection of an intravenous dye and allows quantitative measurements of the retinal vasculature.

OCTA findings of CRAO include diffuse capillary nonperfusion and disruption of the superficial capillary plexus (SCP) and deep capillary plexus (DCP).^[3,4]^ Yang et al^[5]^ showed a reduction of vessel density (VD) in the SCP and DCP, 300 µm around the foveal avascular zone (FAZ), along with an increased FAZ acircularity index and central macular thickness. They showed a correlation between central macular thickness and best-corrected visual acuity (BCVA) but not with other parameters. A case report by Wang et al^[6]^ showed improved vascular parameters on OCTA 9 months after treatment. Hwang et al^[7]^ described a case of CRAO with improvement on OCTA after anterior chamber paracentesis. However, OCTA has limitations, including media opacity, fixation problems, and artifacts such as shadowing, projection, motion, and segmentation.^[8,9]^

Documented treatments of acute nonarteritic CRAO include vasodilators, ocular massage, intraocular pressure (IOP)-lowering therapy, carbogen inhalation, hemodilution, antiplatelet agents, thrombolysis, and/or embolectomy.^[10–13]^ To our knowledge, no longitudinal quantitative OCTA studies have been performed on eyes with CRAO treated with carbogen inhalation. This study aimed to investigate the longitudinal qualitative studies of OCTA characteristics of acute nonarteritic CRAOs treated by carbogen inhalation.

## 2. Patients and methods

This prospective observational study aimed to observe changes in OCTA parameters over time in eyes with acute nonarteritic CRAO treated by carbogen inhalation. The following patients were included: those with acute (within 7 days from onset) nonarteritic CRAO diagnosed between June 1, 2021, and July 31, 2023. The following patients were excluded: patients with ocular comorbidity warranting other acute management, such as giant cell arteritis receiving intravenous corticosteroids; patients previously treated with carbogen inhalation; patients with poor cooperation, dense media opacity, or inability to sit upright; and pregnant women.

CRAO was clinically diagnosed using findings, including cherry-red spots, arterial box-carring, retinal edema, whitening, and/or the presence of an arterial plaque. In cases of uncertain diagnosis, fundus fluorescein angiography and/or indocyanine green angiography were performed.

Each patient was initially treated with ocular massages and IOP-lowering agents immediately after the diagnosis. OCTA measurements were obtained after the treatments, and all patients were admitted to receive carbogen inhalation (a mixture of 5% carbon dioxide and 95% oxygen) at intervals of 15 minutes every 2 hours for 48 hours.

Baseline patient characteristics included age, sex, time from onset (defined as the time from when the patient last noticed normal vision), BCVA (logMAR, logarithm of the minimum angle of resolution), IOP, and any preexisting ocular comorbidity. Subsequently, BCVA and IOP were recorded during admission and at each follow-up visit. The visual acuity of the low vision range was defined using counting fingers = 1.9 logMAR, hand motion = 2.3 logMAR, light perception = 2.7 logMAR, and no light perception = 3.0 logMAR.

This study was approved by the Human Research Ethics Committee of Prince of Songkla University and adhered to the tenets of the Declaration of Helsinki. Written informed consent was obtained from all patients.

### 2.1. OCTA measurements

OCTA measurements were obtained using RTVue XR Avanti AngioVue (Optovue^TM^, CA) with RTVue XR AngioVue 7.0. The image acquisition protocol used was an AngioRetina HD 6 × 6 mm (400 × 400 A scan). If the initial protocol could not be performed, an AngioRetina 3 × 3 mm (304 × 304 A scan) was used. The measurements were taken before admission, at 12 to 24 hours and 36 to 48 hours after administration of carbogen, at 1 to 2 weeks and 4 to 6 weeks of follow-up. There were 25 patients enrolled in the study and 18 patients had completed OCTA measurements at all visits.

**Figure 1.**
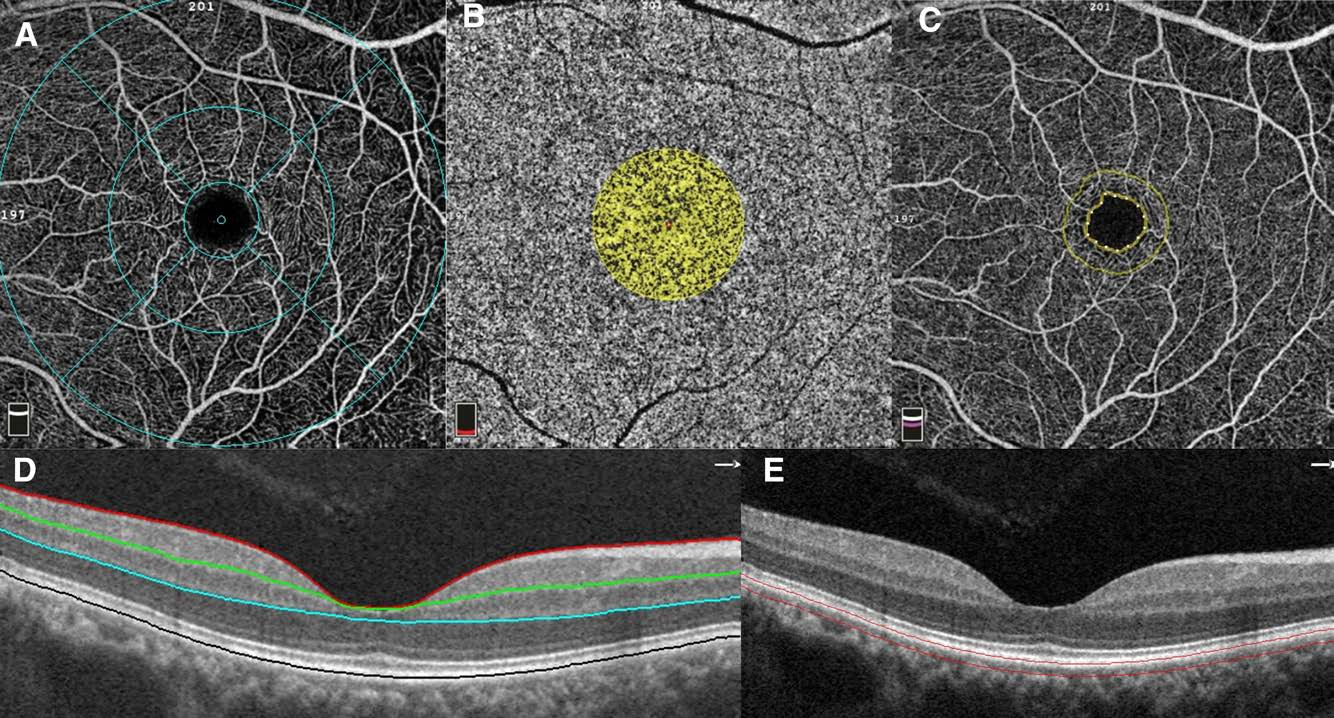
Demonstration of OCTA parameters. (A) Vessel density study area on en-face angiography (OCT C-scan) with an ETDRS subfield overlay. The innermost circle represents the fovea, the middle ring represents the parafovea, and the outermost ring represents the perifovea. (B) Flow area analysis of the outer retina within a 1-mm radius of the fovea. (C) FAZ analysis. The inner dotted ring indicates the FAZ perimeter. The area confined by the FAZ perimeter is the FAZ area. A 300-µm rim area around the FAZ perimeter defines the area of Foveal vessel density (FD-300) measurement. (D) OCT B-scan showing segmentation of the superficial, deep, and outer retina. (E) OCT B-scan showing the choriocapillaris outlined by 2 red lines. ETDRS = early treatment diabetic retinopathy study, FAZ = Foveal avascular zone, OCTA = optical coherence tomography angiography.

Several OCTA parameters (Fig. 1) were collected: VD (%) of the SCP and DCP. The study area was based on the early treatment diabetic retinopathy study subfield including the fovea, parafovea, perifovea, and whole-image area; flow area (mm^2^) within a 1-mm radius of the fovea of the outer retina and choriocapillaris; foveal VD (FD-300, %) defined as VD within 300 µm width rim surrounding the FAZ; FAZ area (mm^2^); FAZ perimeter (mm); and retinal thickness (nm) of the fovea, parafovea, and whole-image area.

Perifoveal and whole-image measurements were omitted from the analysis if the image was acquired using the AngioRetina 3 × 3 mm protocol. Segmentation artifacts were corrected by the researcher before inclusion. Scans with a signal quality indicator of <6 were excluded.

### 2.2. Outcomes

The primary outcome was the comparison of OCTA parameters of the affected eye before and after carbogen inhalation. The secondary outcome was final visual acuity and its correlation with each baseline OCTA parameter in predicting favorable visual outcomes.

### 2.3. Statistical analysis

Discrete variables are presented as percentages and continuous variables as mean ± standard deviation. Continuous variables between the affected and fellow eyes were compared using the Student *t*-test. Continuous variables were compared over time using a repeated measures ANOVA. Correlations between continuous variables were calculated using Spearman rank correlation coefficients. Statistical significance was set at *P* values <.05.

## 3. Results

A total of 25 patients were enrolled in the study and 18 patients had completed OCTA measurements at all visits. Table 1 summarizes the demographic data of the patients.

**Table 1:** Demographic data of participants.

Sex, n (%)	
Male	15 (60.0)
Female	10 (40.0)
Age (year)	
mean ± SD (min, max)	60.68 ± 13.52 (22,79)
Laterality, n (%)	
Right eye	14 (56.0)
Left eye	11 (44.0)
Duration from onset (h)	
Mean ± SD (min, max)	58.64 ± 47.10 (1144)
Ocular comorbidity, n (%)	
Yes	5 (20.0)
No	20 (80.0)
FFA/ICG performed, n (%)	
Yes	4 (16.0)
No	21 (84.0)
Serious adverse event, n (%)	
Yes	0 (0.0)
No	25 (100)
BCVA of affected eye (LogMAR)	
mean ± SD (min, max)	2.20 ± 0.35 (1.32,3)
BCVA of unaffected eye (LogMAR)	
mean ± SD (min, max)	0.16 ± 0.33 (0,1.6)
IOP at onset (mm Hg)	
mean ± SD (min, max)	11.64 ± 3.96 (7,26)

BCVA = best-corrected visual acuity, FFA = fundus fluorescein angiography, ICG = indocyanine green angiography, IOP = intraocular pressure.

### 3.1. VD

The initial OCTA parameters of the affected and unaffected eyes are shown in Table 2. At baseline, the affected eyes showed a significantly increase foveal VD at the SCP (31.61 ± 15.79, 20.89 ± 9.43, *P* = .006) and DCP (47.91 ± 11.78, 34.89 ± 9.56, *P* = .0001) compared to the fellow eye. Contrary to the central subfield findings, a decrease in VD was observed in all other areas of the retina, with statistical significance in all areas.

**Table 2:** Comparison between OCTA parameters in affected and non-affected eyes at baseline

OCTA parameters	Eye		Mean difference (95% CI)	*P*-value
	Affected eye (Mean ± SD)	Non-affected eye (Mean ± SD)	(Affected–non-affected eye)	
Vessel density (%)				
SCP				
Whole image	42.76 ± 7.74	48.03 ± 5.49	−5.58 (−9.59 to −1.56)	.009
Fovea	31.61 ± 15.79	20.89 ± 9.43	9.65 (2.56–16.78)	.006
Parafovea	47.03 ± 8.91	51.32 ± 5.79	−4.12 (−9.01 to 0.78)	.054
Perifovea	42.21 ± 7.83	48.45 ± 5.75	−6.56 (−10.71 to −2.40)	.003
DCP				
Whole image	43.06 ± 6.44	48.35 ± 6.55	−5.43 (−10.10 to −0.76)	.007
Fovea	47.91 ± 11.78	34.89 ± 9.56	13.31 (7.03–19.59)	.0001
Parafovea	43.01 ± 7.88	52.98 ± 5.53	−9.99 (−14.95 to −5.04)	<.001
Perifovea	43.72 ± 6.86	49.73 ± 7.46	−6.06 (−11.11 to −1.00)	.006
Flow area (%)				
Outer retina	0.87 ± 0.54	0.64 ± 0.41	0.23 (-0.12,0.58)	.119
Choriocapillaris	1.37 ± 0.49	1.99 ± 0.15	-0.60 (-0.84, −0.36)	<.001
FD-300 (%)	52.74 ± 10.48	55.27 ± 3.48	-2.60 (-7.11, 1.92)	.269
FAZ area (mm^2^)	0.22 ± 0.11	0.31 ± 0.12	-0.90 (-0.14, −0.04)	.009
FAZ perimeter (mm)	1.77 ± 0.48	2.11 ± 0.43	-0.34 (-0.53, −0.14)	.014
Retinal thickness (µm)				
Whole image	348.13 ± 67.63	279.33 ± 13.60	68.04 (37.98, 98.11)	<.001
Fovea	351.00 ± 108.92	255.38 ± 34.47	92.13 (45.36,138.90)	.0002
Parafovea	409.17 ± 80.29	321.83 ± 18.78	91.09 (54.16,128.02)	<.001

DCP = deep capillary plexus, FAZ = Foveal avascular zone, FD-300 = vessel density 300 µm around the FAZ, SCP = superficial capillary plexus.

The changes in OCTA parameters in the affected eye over time are shown in Table 3. There were no significant changes in VD during the first 48 hours. However, at 1 to 2 weeks a statistically significant decrease in foveal VD was observed in the SCP (−5.68 ± 2.58, *P* = .027) and DCP (−9.92 ± 2.57, *P* <.001). At 4 to 6 weeks, a further decrease in foveal VD was also observed in the SCP (−6.32 ± 2.57, *P* = .014) and DCP (−12.52 ± 2.57, *P* <.001). The whole-image, parafoveal, and perifoveal VDs did not change over time.

**Table 3:** Changes in OCTA parameters over time in the affected eye compared with baseline.

	12–24 h, Mean ± SE (95% CI)*		*P*-value	36–48 h, Mean ± SE (95% CI)*		*P*-value	1–2 wk, Mean ± SE (95% CI)*		*P*-value	4–6 wk, Mean ± SE (95% CI)*		*P*-value
BCVA	−0.27 ± 0.15	(−0.59 to 0.04)	.082	−0.42 ± 0.19	(−0.82 to −0.01)	.045	−0.51 ± 0.18	(−0.90 to −0.12)	.013	−0.58 ± 0.20	(−0.99 to −0.17)	.009
Vessel density												
SCP												
Whole image	−0.28 ± 0.77	(−1.92 to 1.36)	.720	−0.06 ± 1.29	(−2.81 to 2.70)	.966	−0.60 ± 1.70	(−4.23 to 3.03)	.730	−1.43 ± 1.74	(−5.15 to 2.29)	.425
Fovea	0.81 ± 1.97	(−3.40 to 5.01)	.688	−0.74 ± 2.32	(−5.69 to 4.21)	.755	−7.72 ± 3.97	(−16.19 to 0.75)	.071	−7.43 ± 3.82	(−15.56 to 0.70)	.070
Parafovea	1.09 ± 0.85	(−2.89 to 0.71)	.218	−1.77 ± 1.53	(−5.03 to 1.49)	.265	2.76 ± 2.11	(−7.27 to 1.72)	.208	−2.74 ± 2.15	(−7.33 to 1.85)	.222
Perifovea	−0.77 ± 0.89	(−2.67 to 1.14)	.403	−0.41 ± 1.38	(−3.37 to 2.54)	.768	0.07 ± 1.82	(−3.83 to 3.97)	.968	−0.41 ± 1.88	(−4.44 to 3.61)	.829
DCP												
Whole image	−3.81 ± 2.72	(−9.58 to 1.95)	.180	−0.51 ± 1.35	(−3.37 to 2.34)	.709	1.21 ± 1.36	(−1.68 to 4.09)	.389	−1.71 ± 1.46	(−4.80 to 1.39)	.260
Fovea	−3.68 ± 1.66	(−7.23 to −0.13)	.043	−4.64 ± 2.24	(−9.42 to 0.15)	.056	−14.33 ± 3.26	(−21.27 to −7.38)	.001	14.61 ± 2.99	(−20.99 to −8.22)	<.001
Parafovea	−1.74 ± 1.82	(−5.62 to 2.13)	.352	−2.14 ± 2.32	(−7.10 to 2.80)	.371	1.86 ± 2.14	(−2.70 to 6.41)	.399	−1.16 ± 1.52	(−4.40 to 2.07)	.455
Perifovea	−1.41 ± 1.15	(−3.86 to 1.03)	.237	−0.43 ± 1.45	(−3.52 to 2.67)	.773	1.96 ± 1.57	(−1.39 to 5.31)	.232	−1.03 ± 1.65	(−4.54 to 2.49)	.543
Flow area												
Outer retina	−0.15 ± 0.10	(−0.37 to 0.08)	.180	−0.11 ± 0.12	(−0.38 to 0.15)	.369	0.05 ± 0.19	(−0.38 to 0.47)	.817	−0.46 ± 0.23	(−0.96 to 0.04)	.070
Choriocapillaris	0.09 ± 0.04	(0.01–0.19)	.029	0.16 ± 0.04	(0.07–0.25)	.003	0.43 ± 0.11	(0.20–0.66)	.001	0.51 ± 0.12	(0.24 to 0.78)	.001
FD-300	−2.22 ± 2.03	(−6.56 to 2.13)	.293	−2.43 ± 2.09	(−6.92 to 2.05)	.264	−5.40 ± 2.64	(−11.06 to 0.25)	.060	−10.09 ± 2.49	(−15.42 to −4.76)	.001
FAZ area	−0.03 ± 0.02	(−0.07 to 0.08)	.112	−0.01 ± 0.03	(−0.07 to 0.05)	.697	0.07 ± 0.03	(0.07–0.13)	.033	0.02 ± 0.02	(−0.03 to 0.07)	.434
FAZ perimeter	−0.11 ± 0.12	(−0.37 to 0.15)	.385	−0.05 ± 0.15	(−0.36 to 0.27)	.758	0.29 ± 0.15	(−0.04 to 0.61)	.081	0.08 ± 0.12	(−0.18 to 0.34)	.515
Retinal thickness												
Whole image	−2.25 ± 2.82	(−8.27 to 3.77)	.438	−20.19 ± 8.37	(−38.02 to −2.36)	.029	−72.88 ± 15.18	(−105.23 to −40.52)	<.001	−119.44 ± 21.08	(−164.37 to −74.51)	<.001
Fovea	−2.69 ± 6.33	(−16.19 to 10.81)	.677	−26.50 ± 13.24	(−54.72 to 1.72)	.064	−94.75 ± 30.34	(−159.43 to −30.08)	0.007	-112.81 ± 32.43	(-181.94,-43.69)	0.003
Parafovea	−1.31 ± 5.54	(−13.13 to 10.51)	.816	−33.56 ± 12.33	(−59.84 to −7.29)	.016	−103.25 ± 18.53	(−142.74 to −63.77)	<.001	−154.75 to 23.79	(−205.45 to −104.05)	<.001

95% CI = 95% confidence interval, BCVA = best-corrected visual acuity, DCP = deep capillary plexus, FAZ = Foveal avascular zone, FD-300 = vessel density 300 µm around the FAZ, SCP = superficial capillary plexus, SE = standard error of mean.

* Derived from mixed effect random intercept linear regression model.

A comparison of OCTA parameters between the affected and fellow eye at 1 to 2 weeks and 4 to 6 weeks showed no statistically significant differences in the SCP or DCP foveal VD. However, the VD of the whole image, parafovea, and perifovea of both the SCP and DCP significantly decreased at 1 to 2 weeks and 4 to 6 weeks.

### 3.2. Flow area

The choriocapillaris flow area was lower in affected eyes than in unaffected eyes at baseline (1.37 ± 0.49, 1.99 ± 0.15, *P* <.001) (Table 2). An increase compared to baseline in choriocapillaris flow area was seen at 1 to 2 weeks (0.36 ± 0.09, *P* <.001) and 4 to 6 weeks (0.49 ± 0.09, *P* *<*.001; Table 3). A reduction in the flow area in the outer retina, compared to baseline, was also observed at 12 to 24 hours, 36 to 48 hours, and 4 to 6 weeks.

### 3.3. Foveal VD (FD-300), FAZ area, and FAZ perimeter

The FD-300 was slightly higher in the unaffected than in the affected eyes at baseline, but the difference was not significant (55.27 ± 3.48, 52.74 ± 10.48, *P* = .269; Table 2). A progressive decrease in FD-300 of the affected eyes was observed at 36 to 48 hours. Comparison between affected and fellow eyes at 4 to 6 weeks showed significant reduction of FD-300 (42.37 ± 8.76, 54.09 ± 4.53, *P* <.001). The FAZ area and FAZ perimeter were significantly lower than those of the non-affected eye at baseline. Longitudinal observations revealed no significant changes in either parameter.

### 3.4. Total retinal thickness

At presentation, eyes with CRAO showed increased retinal thickness in the whole image (348 ± 67.6, 279 ± 13.6, *P* <.001), fovea (351 ± 108.9, 255 ± 34.5, *P* <.001), and parafovea (409 ± 80.3, 322 ± 18.8, *P* <.001), compared with those in the unaffected eye. This increase was maintained for up to 48 hours. A significant decrease in retinal thickness across all 3 measured domains was seen at 1 to 2 weeks and 4 to 6 weeks. Comparison between the affected and unaffected eyes showed normalization of retinal thickness at 1 to 2 weeks followed by thinning of the retina in all areas at 4 to 6 weeks.

### 3.5. Qualitative assessment

**Figure 2.**
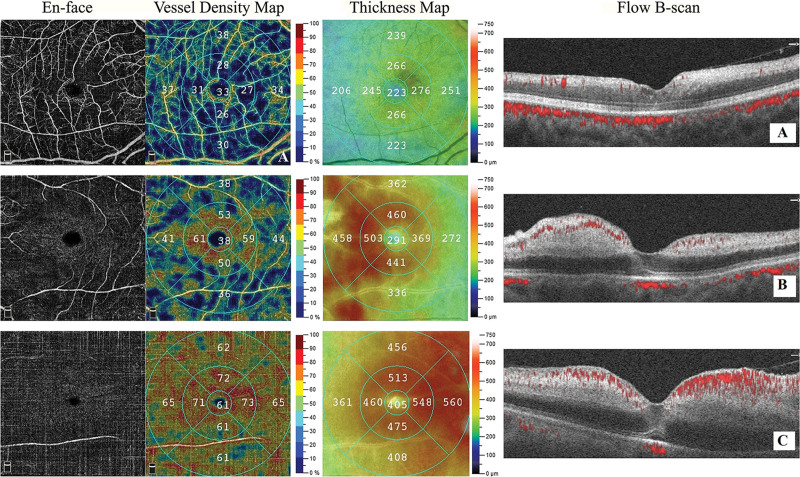
Three characteristic OCTA findings in eyes with central retinal artery occlusion (CRAO) at time of presentation. The leftmost column shows en-face angiography (C-scan) of the superficial retina. The middle left and right columns show the vessel density map of the superficial retina and the total retinal thickness map with color coding, respectively. The rightmost column shows the corresponding flow B-scan, with a red overlay indicating the flow. Top row (A): CRAO with mild retinal edema. En-face angiography shows good preservation of the vessel architecture with capillary dropout. Vessel density is decreased according to the findings seen in en-face angiography. Middle row (B): CRAO with moderate retinal edema. En-face angiography shows fragmentation of large retinal vasculature. Smaller vessels and capillary networks are not identifiable. The vessel density map shows an elevated vessel density correlating with areas of retinal thickening. Bottom row (C): CRAO with severe retinal edema. En-face angiography shows a lack of identifiable vessels. The vessel density is diffusely elevated. Retinal thickening is most pronounced in the left perifoveal, near the optic disc. Flow B-scan shows increased flow signal within the inner retina, shadowing artifacts in the outer retina, and narrowing of the foveal depression. CRAO = central retinal artery occlusion, OCTA = optical coherence tomography angiography.

**Figure 3.**
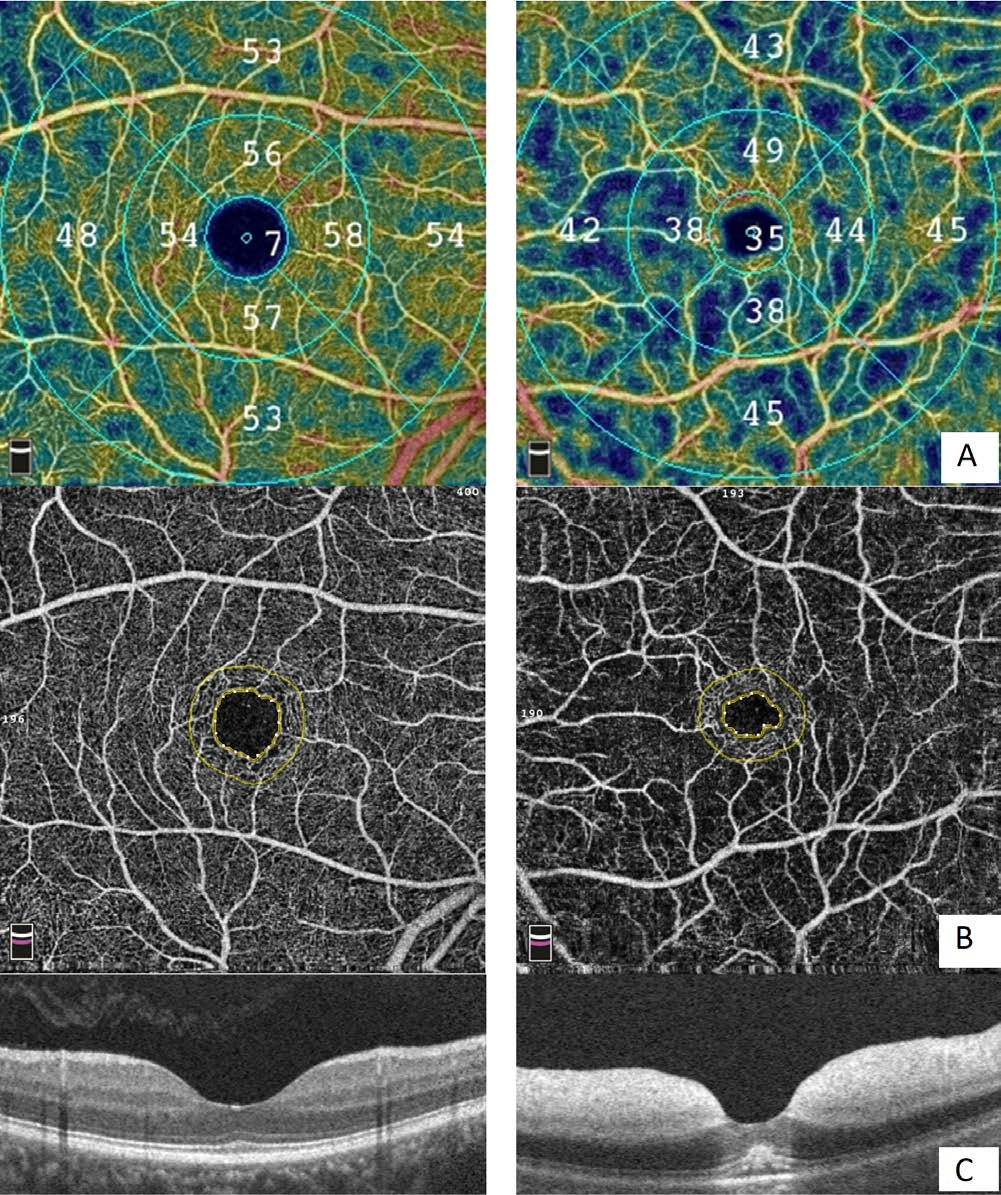
Foveal crowding phenomenon on OCTA. Foveal crowding on OCTA in eyes with CRAO (right column) compared to the normal contralateral eye (left column). The vessel density map shows increased vessel detection in the periphery of the foveal area in the affected eye (top right, A). A reduction in vessel density readings is observed in all areas of the ETDRS subfield except the central subfield. En-face angiography shows FAZ constriction (middle right, B). The corresponding OCT B-scan shows narrowing of the foveal depression due to intrusion of the adjacent edematous retina (lower right, C). ETDRS = early treatment diabetic retinopathy study, OCTA = optical coherence tomography angiography, CRAO = central retinal artery occlusion.

The qualitative assessment of OCTA scans in this study revealed 3 variations in acute CRAOs (Fig. 2). Type 1 represented mild retinal ischemia (14 of 25 eyes). These eyes had a lower degree of retinal thickening and whitening on OCT. An en-face scan showed good preservation of large retinal vessels with areas of capillary dropout. The VD was reduced in all areas, except for the central subfield, in accordance with the results of the study. Type 2 represented moderate retinal ischemia (9 of 25 eyes). These eyes showed a higher degree of retinal edema. An en-face scan showed patchy areas with a complete loss of vessel architecture and abnormally high VD readings located predominantly around the fovea and papillomacular bundle (Fig. 3). Type 3 showed the worse degree of retinal ischemia (2 of 25 eyes). Severe retinal edema with subretinal fluid and severe shadowing artifacts of the outer retina were observed on OCT. The 6 × 6-mm en-face scan showed near-complete loss of the retinal architecture. VD readings were highly elevated in all areas despite the lack of identifiable vessels. At 4 to 6 weeks of follow-up, all eyes showed retinal thinning with capillary dropout and no areas of high VD. Theses falsely high VD readings decreased following the resolution of the retinal edema.

### 3.6. Visual outcome

The initial mean BCVA was 2.20 ± 0.35 LogMAR, whereas the mean final BCVA was 1.7 ± 0.8 LogMAR. Among 25 patients, 9 achieved final BCVA of better than 1.9 LogMAR and 4 achieved final BCVA of better than 0.3 LogMAR. Longitudinal observation showed improvement of BCVA as early as 12 to 24 hours after initiation of carbogen inhalation with maximal improvement of BCVA at 1 to 2 weeks (−0.50 ± 0.11, *P* <.001). Final BCVA changes from baseline in patients presenting within 24 hours from onset and patients presenting after 24 hours showed more favorable visual outcomes in patients with early presentation, but the difference was not significant (−0.77 ± 0.71, −0.31 ± 0.73, *P* = .126).

Spearman correlation between each OCTA parameter and the final BCVA or visual acuity gain showed no strong relationships in either category. No OCTA parameters achieved absolute *R*-value of ≥0.6 although the strongest significant correlation was seen between SCP and DCP foveal VD and final BCVA (*R* = 0.446, *P* = .029, and *R* = 0.491, *P* = .015 respectively).

## 4. Discussion

This study demonstrated the OCTA characteristics of patients with acute nonarteritic CRAO treated with carbogen inhalation. The results showed that eyes with acute CRAO had a lower VD on OCTA than the contralateral eye, except in the foveal area where the VD was initially increased in both the SCP and DCP. We believe that the increased VD at the fovea was due to crowding of the fovea from the intrusion of the edematous inner retina of the parafovea into the central 1-mm zone (Fig. 3), which was reflected in the pronounced decrease in VD at the fovea over time, as retinal edema subsided beginning at 1 to 2 weeks.

The flow area study in the acute phase showed decreased flow in the choriocapillaris compared to that in the fellow eye and a slight increase in flow in the outer retina. The flow area study should be interpreted with caution, as it was affected by shadowing artifacts of the edematous inner retina. This may explain the increase in the choriocapillaris flow area at 1 to 2 weeks and 4 to 6 weeks of follow-up, as the retinal edema gradually subsided. This signal attenuation secondary to high inner retinal reflectivity was consistent with the findings of Baumal^[14]^ and Chen et al.^[15]^ Similar findings were also observed for branch retinal artery occlusion.^[16]^

In the FAZ studies, we found that the FAZ area and FAZ perimeter remained decreased for up to 4 to 6 weeks even after retinal edema and foveal crowding resolved. This is contradictory to the findings in retinal vein occlusion or diabetic retinopathy, which showed enlargement of the FAZ in the presence of ischemic maculopathy.^[17–19]^ A longer follow-up period may be required to fully assess the progression of FAZ characteristics in patients with CRAO.

Regarding the falsely high VD readings, the authors propose that they were generated because of an abnormal low signal-to-noise ratio. Noise is an intrinsic random fluctuation in the reflective measurement between cross-sectional scans. False-positive flow signals can be caused by noise, particularly in areas of low signal.^[20,21]^ Eyes with CRAO may generate both an increase in noise and a decrease in signal in juxtaposition. The hyper-reflective inner retina obscures the underlying vessels, creating an area devoid of contrast. This hyperreflectivity may also cause individual scans of different axes to produce varying reflection amplitudes. This noise is then registered as a flow signal by the split-spectrum amplitude-decorrelation algorithm implemented by Optovue^[22]^ within areas of low signal-to-noise ratio. This increase in VD may explain why the FD-300 was not different between the affected and non-affected eyes when the VD elsewhere was decreased, as the artifactual high VD was most prominent around the fovea. This was also correlated with the reduction in FD-300 as retinal edema resolved.

The LogMAR equivalent of counting finger and hand motions was based on the Freiburg visual acuity test studied by Schulze-Bonsel et al.^[23]^ Light perception and no light perception were based on the imputation by Bach on the same study. Despite the significant visual improvement demonstrated in this study, most patients were still left with severe visual impairment. The mean BCVA at 4 to 6 weeks was 0.48 ± 0.11 LogMAR. This number approximates to visual acuity improvement from hand motion (2.3 LogMAR) to finger count (1.9 LogMAR), correlating with the mean baseline and final BCVA in this study, 2.20 ± 0.35 and 1.7 ± 0.8 LogMAR, respectively.

In conclusion, eyes with acute nonarteritic CRAO showed a generalized reduction in VD, except at the foveal area where high VD was seen initially due to foveal crowding from the edematous inner retina. Excluding the foveal area, no significant change in VD was observed after carbogen inhalation treatment. No baseline OCTA parameters correlated with final visual acuity or visual acuity gain. Pervasive artifacts may limit the use of OCTA as a qualitative tool in eyes with acute CRAO.

## Author contributions

**Conceptualization:** Mansing Ratanasukon.

**Formal analysis:** Pongrapee Atipas.

**Investigation:** Pongrapee Atipas.

**Project administration:** Pongrapee Atipas.

**Resources:** Patama Bhurayanontachai, Pichai Jirarattanasopa, Wantanee Dangboon Tsutsumi, Thada Tantisarasart.

**Supervision:** Mansing Ratanasukon.

**Writing – original draft:** Pongrapee Atipas.
